# The Past and Present of Breast Cancer Resources: A Re-evaluation of the Quality of Online Resources After Eight Years

**DOI:** 10.7759/cureus.28120

**Published:** 2022-08-17

**Authors:** Veronika Killow, Julia Lin, Paris-Ann Ingledew

**Affiliations:** 1 Radiation Oncology, British Columbia Cancer Agency, Vancouver, CAN; 2 Family Medicine, University of Calgary, Calgary, CAN

**Keywords:** oncology websites, internet information, patient information, online resources, cancer websites, patient education, breast cancer

## Abstract

Background and objective

The internet has become a major resource of information for cancer patients. However, the quality of these resources is variable, and a better understanding is needed to guide physicians as to how to best support patients in their online searches. We previously evaluated the quality of online breast cancer resources in 2011. Nearly a decade later, we aimed to assess the present quality of online breast cancer-related information and to compare our current analysis with data collected in 2011.

Methods

A list of 100 breast cancer websites was systematically compiled using meta-search engines Yippy and Dogpile and the search engine Google using the search term “breast cancer”. Content accuracy and quality markers, including authorship, attribu­tion, currency, site organization, and readability were assessed by using a previously validated standardized rating tool. Results were analyzed using descriptive statistics and Fisher’s exact test. The same strategy was used in both 2011 and 2019.

Results

When comparing 2011 data to the current one, 27% of websites had been updated in the previous two years in 2011 compared to 65% in 2019 (p<0.00001). Both data sets remained similar in terms of website disclosures and objectivity. Only 30% of websites analyzed in 2019 used two or more reliable sources, while 63% had no reliable sources or no sources cited. From 2011 to 2019, resources with readability above grade 12 increased from 4% to 30% (p<0.0001), while websites offering educational support rose from 8% to 35% (p<0.0001). In 2019, treatment and etiology/risk factors were the most accurately covered areas (64% and 63% of websites, respectively). In 2011, 63% of websites were found to be globally accurate. Prognosis coverage increased from 18% to 33% from 2011 to 2019 (p=0.02). In 2019, survivorship was also evaluated and found to be covered in only 24% of resources.

Conclusion

Over the past eight years, there have been variable changes in the quality of online breast cancer resources. Promisingly, websites are being updated more frequently and the educational support offered is expanding. Furthermore, there has been significant improvement in the coverage of prognosis, although this requires further progress. Unfortunately, websites are becoming increasingly challenging to understand for the average patient, and coverage of survivorship is lacking. Our study provides vital information to healthcare providers on these trends in online breast cancer resources and how to best support patients in their internet searches.

## Introduction

In 2020, 19.3 million new cases of cancer were diagnosed worldwide [1]. It is estimated that 225,800 Canadians were diagnosed with cancer and 83,300 died from the disease in 2020 alone [2]. Of these cases, breast cancer is the most commonly diagnosed cancer in women [1], accounting for nearly 25% of new cancer cases [2]. Many cancer patients are turning to the internet to seek out information with regard to their treatment and prognosis [3-4]. Studies show that the prevalence of cancer patients who use the internet to look for cancer-related information ranges from 77% to 89% [5-7], and it appears to be on the rise [5]. Furthermore, internet searches have been shown to impact patient decision-making, with one study reporting that 53% of patients had their treatment decisions influenced by their online searches [8]. However, the quality of online resources is variable [9], and a better understanding is needed to guide physicians as to how to best support patients in their online searches. In this study, we aimed to assess the quality of online resources for breast cancer patients and examine how this has changed with time. We previously evaluated the quality of online breast cancer resources in 2011. Now, nearly a decade later, we aimed to assess the quality of online breast cancer-related information and compare our current analysis to data collected in 2011 by using the same comprehensive rating tool.

This article was previously presented as a poster at the 2020 Canadian Association of Radiation Oncology Annual Scientific Meeting on September 24, 2020; the 2020 American Society for Radiation Oncology Annual Meeting on October 27, 2020; and the 2021 San Antonio Breast Cancer Symposium on December 9, 2021.

## Materials and methods

A list of breast cancer websites was generated by inputting the search term “breast cancer” into the meta-search engines Dogpile and Yippy and the search engine Google. The meta-search engines Yippy and Dogpile search Google, Bing, and Ask.com as part of their search [10], and therefore are representative of a greater number of search results potentially found by patients using their search engine of choice, with a heavier weight given to Google, the most popular search engine [11]. A list of 500 websites was documented based on the results from each of the search engines. Pre-determined exclusion criteria were then applied to exclude websites requiring subscriptions, those not in English, duplicates, and those without functional links. Each website was assigned a value based on its average rank order appearance on the three search engine results. The first 100 websites on the combined list were termed “100 websites”. This list was first generated and analyzed in 2011. A second list was generated in 2019. The same methodology was used in 2011 and 2019 to conduct the study.

In both 2011 and 2019, a previously validated standardized rating tool was used to evaluate the list of the “100 websites”. The tool was iteratively developed and based on JAMA, the Health on the Net Foundation Code of Conduct (HONcode), DISCERN, and a detailed review of other pre-existing approaches and resources to evaluate web-based resources [12]. The various components of this tool have been validated elsewhere [13-16].

This tool was used to assess content, accuracy, and quality markers, including website affiliation, authorship, attribution, disclosure, currency, interactivity, site organization, and readability. Affiliations were assigned initially based on the website domain (i.e. “.com” for commercial, “.org” for nonprofit, “.gov” for government, “.edu” for educational), and then verified manually. Websites affiliated with a registered charity were categorized as nonprofit; websites published by a national, provincial, state, or municipal authority were classified as government and educational websites were categorized as such if they were published by a post-secondary institution. The remaining websites were classified as commercial. Authorship was assessed based on whether websites identified an author, their affiliations, and their credentials. Attribution was determined by looking for stated sources of published information and the number of sources used. Sources cited were evaluated for reliability, where the use of journal articles, peer-reviewed sites like UpToDate, textbooks, and academic and government sites was considered reliable. The disclosure aspect was evaluated by verifying the website’s ownership and whether sources of advertising were clearly stated. Regarding site organization, we looked for structural tools such as headings, subheadings, diagrams/pictures/tables, hyperlinks, and the absence of advertising. Accountability was assessed by manually checking for the presence of author information, attribution, disclosure, currency, and external links.

Websites were evaluated for interactive elements, including educational support (e.g., modules and workshops), search engines, audio or video support, discussion boards, and a medical professional’s contact information based on the adaptation of the Abbott’s scale [17]. The readability was established using readable.com’s assessment tool to determine the Flesch-Kincaid grade level and Simple Measure of Gobbledygook (SMOG) Index. The introduction and treatment sections of each website were directly inputted into readable.com, when possible, to generate a readability score. If these sections were missing, the section on etiology/risk factors was preferred, followed by the diagnosis section.

Content and accuracy were evaluated based on the materials deemed to be necessary for a patient’s understanding of breast cancer by a content expert. Breast cancer information from the National Comprehensive Cancer Network (NCCN) and UpToDate were reviewed and summarized by the research assistant to create a consensus document of key information to be used as a consistent benchmark for evaluating information accuracy. These peer-reviewed resources were chosen for consistency with previous similar studies [18-22]. The document was reviewed by the research assistant and a practicing breast cancer oncologist to determine the level of detail required under each heading to obtain a score of not accurate, mostly accurate, or completely accurate. Websites were considered objective if they contained verifiable facts without bias or persuasive language. The results were analyzed using descriptive statistics, Fisher’s exact test, an unpaired t-test for categorical variables, and the two-sided Welch's t-test.

Between 2011 and 2019, there were changes in the tool due to the iterative use of the tool, consistent with the principles of design-based research [12,23]. In 2011, websites were evaluated for overall quality based on the overall accuracy of website information, whereas in 2019, content accuracy was assessed for each content section, i.e., the definition, incidence, etiology, detection, treatment, prognosis, and prevention sections. In 2019, some additional data were collected, including whether websites were able to answer general and personal health questions when contacted. In 2019, websites with available contact information were sent a general and a personal health question to determine responsiveness to patients. The general question used was “Are family members of people with ductal breast cancer at higher risk for getting breast cancer?”. For the personal question, the contacts were asked “I am a patient that was treated for an early ductal breast cancer (lumpectomy, radiation, and endocrine therapy). How long will I remain on endocrine therapy?”. Website responses were evaluated for accuracy of response and time to response. A response contradicting the information on UpToDate or NCCN was considered inaccurate. These responses were evaluated separately from the website scoring system.

## Results

**Figure 1:**
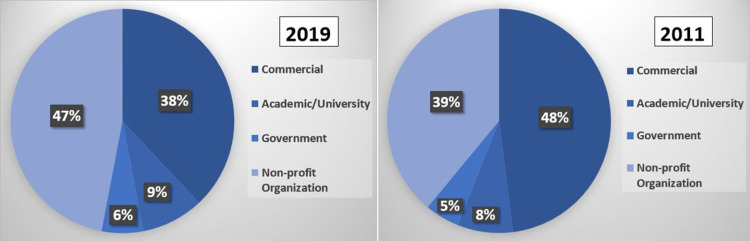
Website Affiliations in 2019 and 2011

Summaries of the different characteristics of websites in 2011 and 2019 are shown in Figure 1. There were more commercial websites in 2011 compared to 2019 (48% vs. 38%), and fewer non-profit websites (39% vs. 47%). There were similar proportions of academic and government websites (8% vs. 9%, and 5% vs. 6%, respectively), as shown in Table 1.

**Table 1:** Summary of Website Results in 2011 and 2019, Listed as Percentage of Websites

Measured Feature		2019	2011	P-value
Affiliation	Commercial	38%	48%	1.0
	Non-Profit	47%	39%	
	Academic	9%	8%	
	Government	6%	5%	
Authorship	Author Identified	45%	34%	0.15
	Affiliations Identified	26%	20%	0.40
	Credentials Identified	33%	26%	0.35
Attribution	Information Cited	37%	39%	0.88
	At Least 3 Sources Used	29%	24%	0.52
	Cited >1 Reliable Sources	30%	29%	0.99
	Cited 1 Reliable Source	7%	8%	
	Cited No Reliable Sources	63%	62%	
Disclosure	Disclosure Present	76%	86%	0.10
Currency	Creation Date Provided	30%	73%	<0.00001
	Modification Date Identified	50%	44%	0.48
	Update Date Within 2 Years	65%	27%	<0.00001
	Update Date Within 4 Years	68%	37%	<0.01
Links	Two or More External Links	42%	58%	0.03
	One External Link	5%	9%	0.41
	No External Links	53%	33%	0.007
	>50% of Links Accessible	45%	62%	0.02
	<50% of Links Accessible or No Links	55%	38%	0.02
Interactivity	Search Engine Present	82%	79%	0.72
	Audio/Visual Support Available	38%	32%	0.46
	Discussion Board or Forum Available	16%	14%	0.84
	Queries to Website Supported	63%	41%	0.003
	Education Support Offered	35%	8%	<0.00001
Readability	Reading Level Above Grade 6	100%	93%	0.01
Global Accuracy	Completely Accurate	28%	63%	1.0
	Mostly Accurate	48%	37%	
	Mostly Not Accurate	24%	0%	
Objectivity	Website Objective With No Bias/Opinion Present	86%	83%	0.70
Queries to Medical Professional Supported	Email Response Within 48 Hours	21%	n/a	n/a
	Email Response Within 7 Days	6%	n/a	n/a
	No Email Response	41%	n/a	n/a
	Unable to Contact	39%	n/a	n/a
General Question Website Response	Accurate Response Provided	12%	n/a	n/a
	Provided Links Only	4%	n/a	n/a
	Unable to Provide Advice	11%	n/a	n/a
Personal Question Website Response	Accurate Response Provided	10%	n/a	n/a
	Provided Links Only	4%	n/a	n/a
	Unable to Provide Advice	13%	n/a	n/a

**Figure 2:**
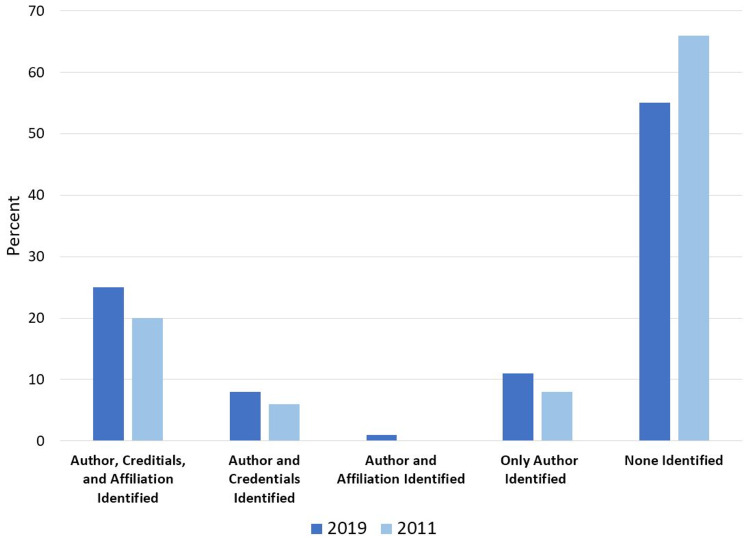
Authorship Identification in 2019 and 2011

In 2011, authors with author affiliations and credentials were identified in 20% of websites, while 66% had no author, affiliation, or credentials listed (Figure 2). This was similar to 2019, where authors, author affiliations, and credentials were identified in 25% of websites, while 56% of websites did not identify any of the three categories.

In 2011 and 2019, there was a similar number of websites citing their sources of information (39% vs. 37%). In both 2011 and 2019, 30% of websites were found to use two or more reliable sources; in both years, 63% of websites used no reliable sources or had no sources cited. The most commonly cited sources in 2011 were non-profit websites and journal articles. This trend continued in 2019. In 2011, 86% of websites disclosed site ownership, sponsorship, and advertising. In 2019, this number dropped to 76% but was not found to be statistically different from 2011 (p=0.1).

In 2011, 73% of websites identified the date of website creation, compared to only 30% of websites in 2019 (p<0.0001). In 2011, 27% of websites had been updated within two years, compared to 65% in 2019 (p<0.00001). In 2011, 67% of websites had been updated over four years ago or did not provide information on the last update, compared to 32% in 2019 (p<0.01). In 2011, 33% of websites provided no non-advertisement external links compared to 53% in 2019. There were more websites with accessible links in 2011 compared to 2019 (62% vs. 45%).

When evaluating website interactivity, access to an in-website search engine, audio or visual support, and discussion board or forum were similar in 2011 and 2019 (p=0.72, p=0.46, p=0.84, respectively). In 2011, 41% of websites provided a way to contact a medical professional, which significantly increased to 63% in 2019 (p<0.003). The number of websites providing educational support in 2019 was also significantly higher when compared to 2011, at 35% compared to 8% (p<0.0001). When comparing site organization (headings, subheadings, pictures and tables, hyperlinks, and no advertisement) between 2011 and 2019, there were no significant changes in the number of tools used (p=1).

In 2019, out of the 63 websites that displayed contact information for a medical professional, only 27 (43%) responded to an emailed question, with 21 (33%) responding within 48 hours, and six (10%) responding within seven days. Of those that replied, 12 websites accurately answered the general question, four provided links only, and 11 stated they were unable to provide advice. Ten websites accurately answered the personal question, four provided links only, and 13 stated they were unable to provide advice. None of the websites provided inaccurate or harmful advice for either question.

**Figure 3:**
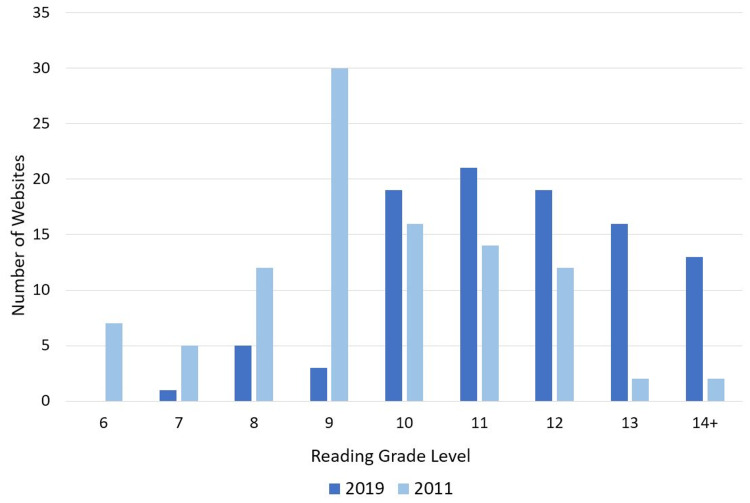
Website Reading Level by Grade in 2019 and 2011

When comparing readability, 2011 data had 12% of websites at the elementary school level (Grade 7 or below), 84% of websites at the high school level (Grade 8-12), and 4% of websites at the university level or higher (Figure 3). In 2019, there was a statistically significant increase in the reading grade level from 2011 (p<0.001), with only 1% of websites at the elementary school level, 67% at the high school level, and 30% at the university level. The SMOG index also significantly increased from 2011 (p=0.01), while the Flesch-Kincaid Readability score, where a high score indicates better readability, was statistically higher in 2011 (p<0.0001).

**Figure 4:**
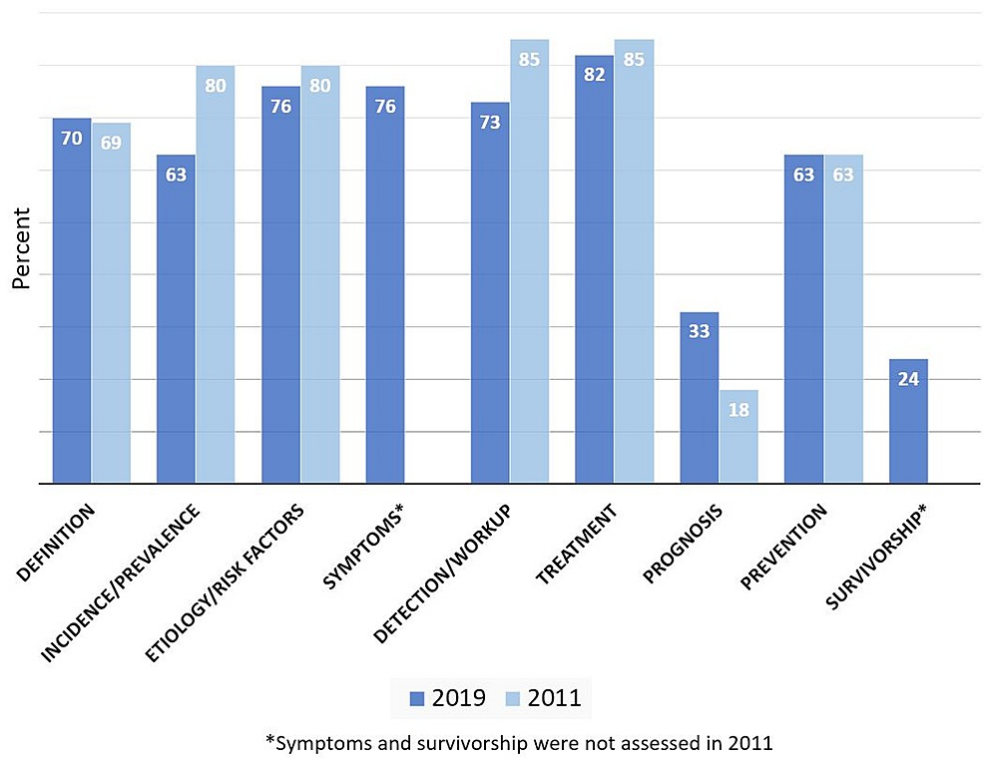
Website Content Coverage by Topic in 2019 and 2011

In terms of website coverage of different topics (definition, prevalence, etiology, detection, treatment, prognosis, and prevention), breast cancer definition, etiology, treatment, and prevention were covered similarly in both 2011 and 2019 (Figure 4). Prevalence was covered by 80% of websites in 2011 compared to 63% in 2019 (p=0.01), detection was covered by 85% of websites in 2011 compared to 73% in 2019 (p=0.055), and prognosis was covered by 18% in 2011 compared to 33% in 2019 (p=0.02). In 2019, survivorship (a new category added in 2019) was covered by 24% of websites. Symptoms were also a new category in 2019 and were covered by 76% of websites.

In 2011, 63% of websites met the criteria for being completely accurate, while in 2019, only 28% met these criteria. In 2011, 37% of websites were mostly accurate, and in 2019, 48% of websites met the same criteria. In 2011, zero websites were deemed mostly not accurate, but in 2019, this number rose to 24%.

In 2019, a categorical assessment was done to assess accuracy, which found that etiology and treatment had the highest accuracy, with 63% and 64% of websites having completely accurate information, respectively. Survivorship and prognosis were the least accurate, with only 7% and 9% of websites being completely accurate, respectively.

Objectivity between 2011 and 2019 remained similar, with 83% of websites having no apparent bias in 2011 compared to 86% of websites in 2019.

## Discussion

Despite the internet continuing to grow in popularity as a resource for patients, there has been limited data on the quality of resources, especially as to how online resources are changing over time. This information is especially important as misinformation becomes a major concern. Furthermore, the rapid evolution of the internet requires ongoing reassessment.

At least two prior studies have looked at the quality of breast cancer resources. One previous study by Nghiem et al. in 2016 looked at 26 breast cancer websites using three search engines and evaluated them by using the DISCERN Plus tool, HONcode, and JAMA benchmarks [24]. As with our study, they assessed authorship, attribution, disclosure, and currency. They found that 35% of websites were created by qualified medical professionals or a clear statement was made to state otherwise. Similarly, in our study, the number of websites meeting these criteria was 26% in 2011 and 33% in 2019. Using JAMA benchmarks, Nghiem et al. found that 77% met the disclosure benchmark. Similarly, in our study, 86% and 76% of websites from 2011 and 2019, respectively, disclosed ownership of the site and sponsorship. When it came to attribution, Nghiem et al. found that 62% of websites provided clear references for their information. In our study, 61% and 63% of websites in 2011 and 2019, respectively, cited sources of information. Interestingly, unlike our study, Nghiem et al. found that among their websites, 81% met the JAMA currency benchmark, which required websites to provide dates for when information was posted and updated. In our study, these numbers were hovering near 50% in both 2011 and 2019 for identifying the date of modification, and 73% and 30% for 2011 versus 2019, respectively, for identifying a date of creation. Of note, Nghiem et al. did not use meta-search engines, used a limited number of websites, and did not review data trends over time.

Another study by Arif and Ghezzi in 2018 specifically focused on online resources related to breast cancer treatment by reviewing 200 websites on Google UK [25]. This review focused on assessment using JAMA score, HONcode certification, and readability. Their websites had a mean readability grade of 8.5, which was calculated using the same online readability tool as in our study. This was much lower than the mean of 11.6 found in our study. In line with our study, only 35% of websites mentioned the source of their information, while only 36% reported authorship. Like our study, they concluded that the quality of information relating to breast cancer on the internet is variable.

Our findings suggest that breast cancer websites may be evolving to include more frequent updates, with approximately two-thirds of websites in 2019 updated in the last two years, compared to less than one-third in 2011. This is a reassuring trend as cancer research continues to progress and change at rapid rates. Similar studies reviewing online resources for other malignancies found less frequent updates, with one study finding 50% of websites updated in the last two years [18], while two other similar studies showed that only 20-32% of websites met these criteria [20-21]. This perhaps suggests that while breast cancer resources may be exhibiting more frequent updates, this trend may not apply to other malignancies.

In terms of website authorship, the acknowledgment of authors has remained poor and has not significantly improved over the last eight years. In similar recent studies on other malignancies, authorship identification was also found to be low, ranging from 26% to 40% of websites identifying an author [18,20-22]. Authorship is a key aspect in evaluating resources for their accountability.

Unfortunately, our data suggest that the readability of websites has substantially worsened, with 30% of websites now reading at above grade 12 level, compared to only 4% in 2011. Several similar studies evaluating site-specific cancer resources (i.e., pancreatic, thyroid, and cervical cancers) found that 18-22% of websites had an above grade 12 reading level [18,20,22]. A paper evaluating reading levels in online glioblastoma resources found that 63% of websites had readability above grade 12 [21]. Another study by del Valle et al. evaluated the readability levels of websites discussing preventative mastectomies using the SMOG tool [26]. When looking at the top 10 English language websites, the SMOG reading grade level was found to be 14.69, ranging from 12.6 to 16.2, again suggestive of the very high readability levels overall. Basch et al. specifically looked at the readability of 100 breast cancer websites using five different tests, including the Flesch-Kincaid grade level [27]. They found that 45% of websites were at reading levels above grade 10, and only four websites were below grade 6 level. This suggests a lower average reading level compared to our study, where 71% of websites were found to have reading levels above grade 10. None of the 2019 websites reviewed in our study were below the recommended grade 6 reading level [28]. Based on the studies above, there may be some variation in the complexity of material depending on the cancer site discussed. However, the data suggest that the complexity of online resources is frequently too convoluted for many patients accessing these resources.

Our study had several limitations. One major limitation of this study was its focus on resources written in English. Additional studies are necessary to assess whether online resources in other languages are similar in quality and content. Furthermore, the searches were carried out from a single location, which may result in location-based results from the search engines. However, we attempted to mitigate this bias by using meta-search engines’ incognito mode. Further studies may be necessary to account for the influence of geographic location on top search results and assess its impact on patient searches. Our study evaluated websites based on their coverage of numerous topics, whereas some websites may be designed to provide information on specific aspects of breast cancer. Thus, when evaluating “overall scores”, there may be some difficulty in interpretation, as specific sites might not have scored as high on content. In the future, our tool may be modified to factor in the purpose of each website to ensure that a website is fairly evaluated for both content and quality. When considering readability, our study specifically focused on the introductory and treatment sections to assess this, and therefore the result may not always accurately represent the website’s true readability level. Of note, this study did not evaluate social media pages, which patients may use to seek information. In the future, the tool may be modified to assess this aspect of online information. A strength of this paper was its strong-evidence-based methodology, achieved by the use of a robust and previously validated standardized rating tool. The number of websites evaluated was quite large, which provided a more thorough overview of the landscape of resources available. The use of meta-search engines to conduct our search and the use of incognito mode created a more representative list of search results potentially found by patients.

## Conclusions

It is important for healthcare professionals to be aware of potential gaps in resources their patients are accessing so that appropriate discussion can be initiated to address these gaps. Furthermore, healthcare professionals can play an active role in recommending trusted resources and educating patients on the benefits and potential pitfalls of online resources. Over the past eight years, there have been variable changes in the quality of online breast cancer resources. Promisingly, websites are being updated more frequently and the educational support offered is expanding. Furthermore, there has been significant improvement in the coverage of prognosis, although this requires further progress. Unfortunately, websites are becoming increasingly challenging to understand for the average patient with respect to readability, and coverage of survivorship is lacking. Our study provides pertinent information to healthcare providers on these trends in online breast cancer resources and how to best support patients in their internet searches.
